# Research hotspots and trends in retinopathy of prematurity from 2003 to 2022: a bibliometric analysis

**DOI:** 10.3389/fped.2023.1273413

**Published:** 2023-10-03

**Authors:** Yulin Zhang, Xiaoyan Chai, Zixin Fan, Sifan Zhang, Guoming Zhang

**Affiliations:** ^1^ Shenzhen Eye Hospital, Jinan University, Shenzhen Eye Institute, Shenzhen, China; ^2^ Department of Biology, New York University, New York, NY, United States

**Keywords:** retinopathy of prematurity, research trends, research hotspots, bibliometric analysis, citespace

## Abstract

**Background:**

In order to understand the research hotspots and trends in the field of retinopathy of prematurity (ROP), our study analyzed the relevant publications from 2003 to 2022 by using bibliometric analysis.

**Methods:**

The Citespace 6.2.R3 system was used to analyze the publications collected from the Web of Science Core Collection (WoSCC) database.

**Results:**

In total, 4,957 publications were included in this study. From 2003 to 2022, the number of publications gradually increased and peaked in 2022. The United States was the country with the most publications, while Harvard University was the most productive institution. The top co-cited journal PEDIATRICS is published by the United States. Author analysis showed that Hellström A was the author with the most publications, while Good WV was the top co-cited author. The co-citation analysis of references showed seven major clusters: genetic polymorphism, neurodevelopmental outcome, threshold retinopathy, oxygen-induced retinopathy, low birth weight infant, prematurity diagnosis cluster and artificial intelligence (AI). For the citation burst analysis, there remained seven keywords in their burst phases until 2022, including ranibizumab, validation, trends, type 1 retinopathy, preterm, deep learning and artificial intelligence.

**Conclusion:**

Intravitreal anti-vascular endothelial growth factor therapy and AI-assisted clinical decision-making were two major topics of ROP research, which may still be the research trends in the coming years.

## 1. Introduction

Retinopathy of prematurity (ROP) is a vascular proliferative disease that primarily occurs in the immature retina of preterm, low-birth-weight infants, leading to permanent visual impairment (1). Its incidence has been increasing gradually over the past 20 years in many countries and regions, probably due to the improved survival of neonates and increased ROP screening (2–4). So far it has been considered the leading cause of blindness in children worldwide, resulting in growing burden on healthcare system (5). In order to get a more comprehensive understanding about the research progress of ROP diagnosis and treatment, we performed a bibliometric analysis in this study by using the CiteSpace.

Bibliometric analysis focuses on the quantitative analysis of publications by using statistical methods, describing the research trend in a field and the relationship between published information (6). As a tool for bibliometric analysis, the software CiteSpace can further support the visualization of cooperation, co-citation and co-occurrence networks, thereby giving more insights into the frameworks and rules of a certain knowledge domain (7). Therefore, in this study, we used CiteSpace not only to summarize the evolution of past ROP researches, but also to explore the research frontiers.

## 2. Materials and methods

### 2.1. Data sources and collection

All the literature were searched from the Web of Science Core Collection (WoSCC) database in SSCI and SCI-EXPANDED. The search strategy was TS = (retinopathy of prematurity) OR Tl = (retinopathy of prematurity) OR AB = (retinopathy of prematurity) OR AK = (retinopathy of prematurity) OR KP = (retinopathy of prematurity). The inclusion criteria were as follows: the year of publication was between 2003 and 2022; the classification of language was English; and the type of literature was article or review. The date of data collection was on May 28, 2023.

### 2.2. Statistical analysis

Citespace 6.2.R3 was used to remove duplicates before analyzing the data. The time slice length was set to 1 year and the links strength was calculated by cosine algorithm with the scope within slices. The processes of data mining and visualization were as follows. Firstly, the cooperation relationships between countries, institutions and authors were analyzed and visualized with the threshold set to top 30. The centrality of a node was calculated according to the number of times that it acted as the shortcut between two other nodes. A higher centrality to some extent represented a more critical position in relationship. Secondly, co-citation analysis of authors, journals and references were done with top 30 as a standard. It was defined that if two articles appeared together in the references of a third citing article, they formed a co-citation relationship. The network of co-cited references was visualized and then clustered by the keyword list of citing references. The log-likelihood ratio (LLR) algorithm was used for label selection and clustering. Clusters were considered of high reliability with silhouette value larger than 0.7. Thirdly, the keyword analysis was performed by counting their co-occurrence in the same literature. A modified g-index was selected with the scale factor k set to 25 and a timeline map was drawn for visualization. Finally, citation bursts were detected in references and keywords, reflecting the research hotspots of different stages. Top 25 burst nodes were shown and sorted by the beginning year of burst.

The visualization results were represented by nodes and lines. Larger nodes indicated more publications or citation counts. Lines between nodes suggested the cooperative or co-cited relationship. Colors of nodes and lines corresponded to the years of publication or first citation. Nodes with purple outer circles were those with high centrality.

## 3. Results

### 3.1. Global distribution of publications

**Figure 1:**
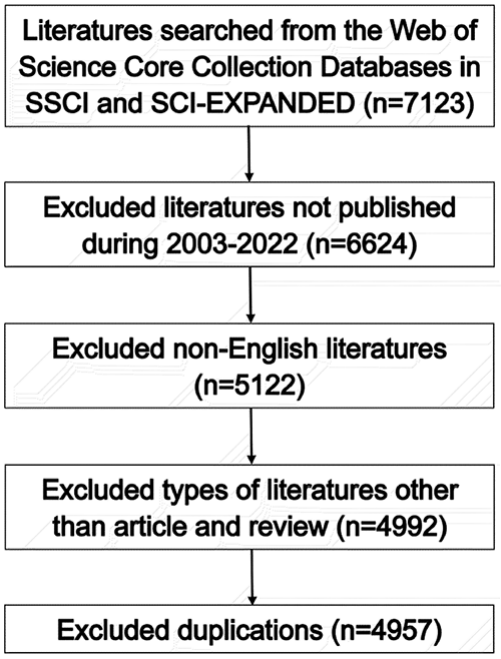
Flowchart of the inclusion and exclusion processes.

**Figure 2:**
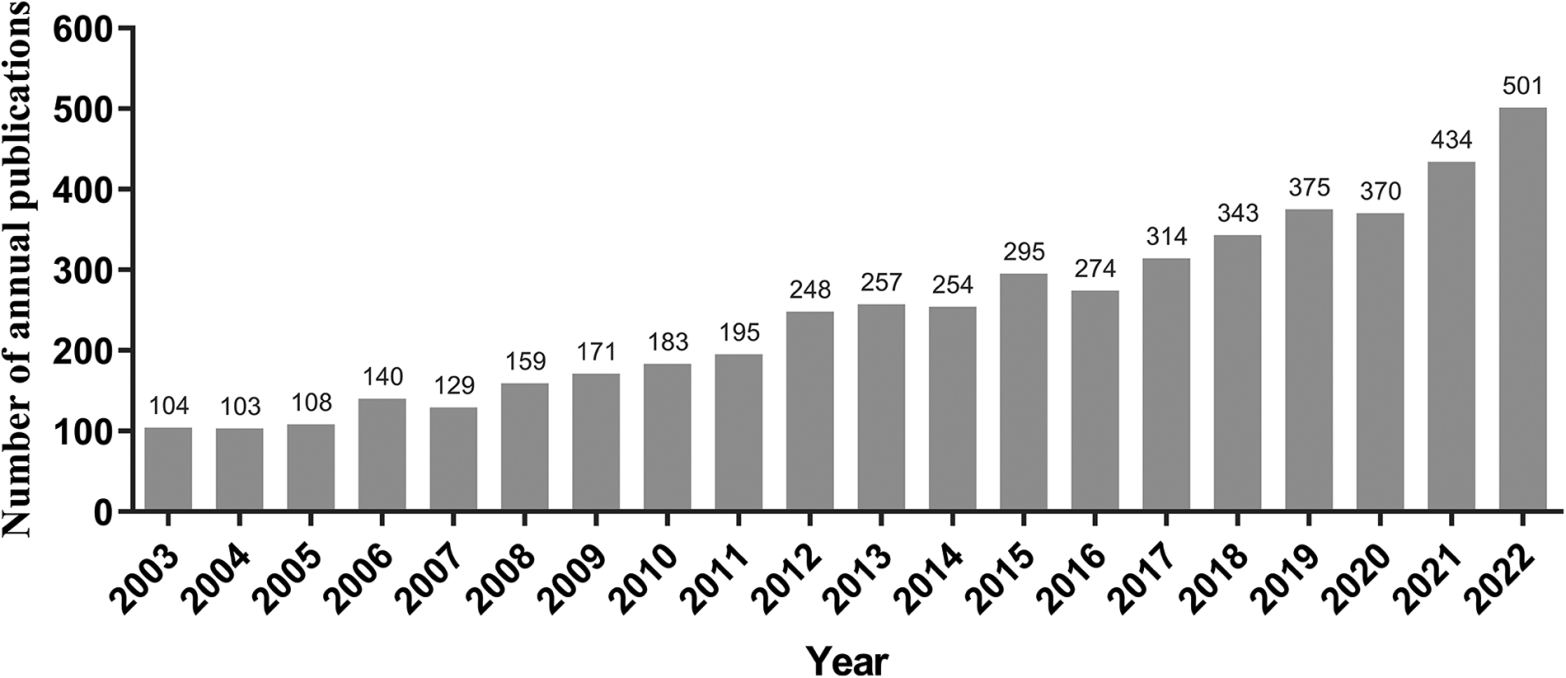
The annual publication in ROP from 2003 to 2022.

**Figure 3:**
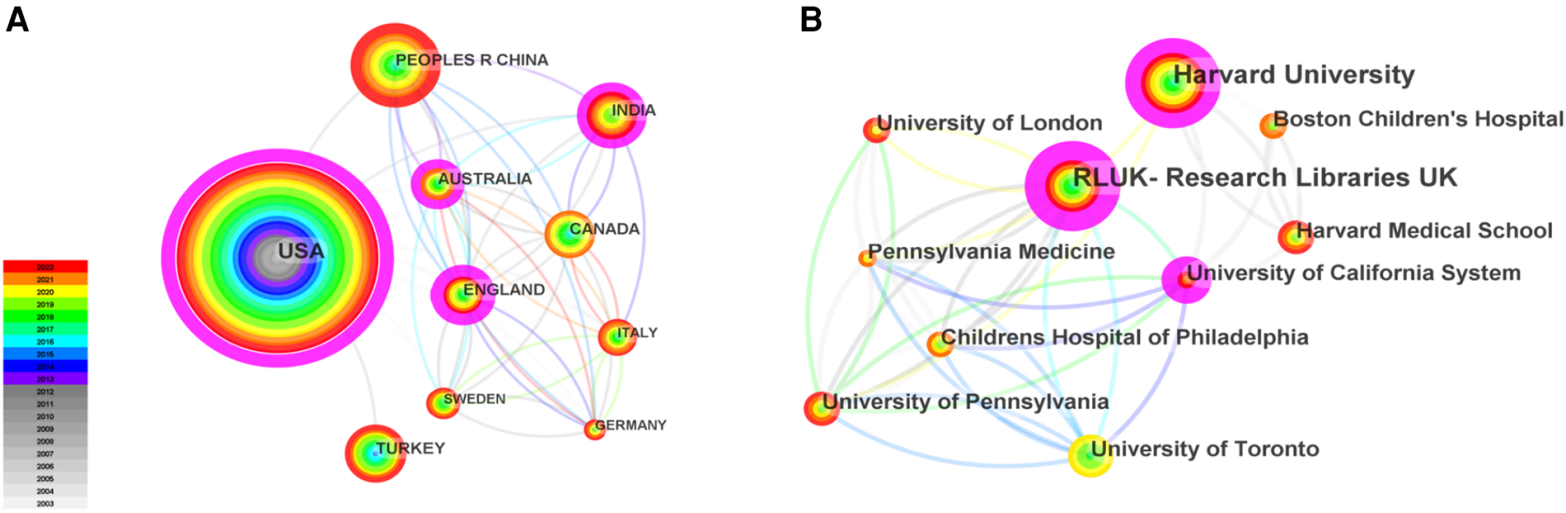
The cooperation network between the top 10 productive countries (**A**) and institutions (**B**).

As shown in Figure 1, totally 4,957 publications were input for further bibliometric analysis, and the number of publications was listed by year in Figure 2. In 2003, there were only 104 published papers relevant to ROP globally, and the number gradually increased in fluctuations, reaching its maximum by 2022 (*n* = 501, 10.11%). The top 10 countries ranked by number of publications were listed in Table 1, and their cooperation network was presented in Figure 3A. It could be seen that the United States contributed the most (*n* = 1891, 38.15%) with highest centrality (0.55) in the network, followed by China (*n* = 545, 10.99%) and Canada (*n* = 326, 6.58%). As for the top 10 institutions (Table 2 and Figure 3B), Harvard University ranked the first in the number of publications (*n* = 296, 5.97%), while Research Libraries UK possessed the top centrality (0.22) in cooperation. Furthermore, by conducting a co-citation analysis, the top 10 journals were shown in Table 3. Nine out of 10 of the journals came from the United States, and PEDIATRICS with 3,033 citation counts ranked first.

**Table 1:** The top 10 countries listed by number of publications.

Rank	Country	Publications	Centrality
1	USA	1891	0.55
2	PEOPLES R CHINA	545	0.01
3	CANADA	326	0.08
4	ENGLAND	311	0.28
5	INDIA	307	0.12
6	TURKEY	286	0.00
7	AUSTRALIA	266	0.14
8	SWEDEN	225	0.06
9	GERMANY	220	0.06
10	ITALY	196	0.02

**Table 2:** The top 10 institution listed by number of publications.

Rank	Institution	Publications	Centrality
1	Harvard University	296	0.14
2	RLUK- Research Libraries UK	263	0.22
3	Harvard Medical School	226	0.08
4	Boston Children’s Hospital	191	0.05
5	University of Pennsylvania	168	0.04
6	Pennsylvania Medicine	146	0.05
7	University of Toronto	145	0.03
8	University of California System	143	0.13
9	Childrens Hospital of Philadelphia	136	0.07
10	University of London	129	0.03

**Table 3:** The top 10 co-citation journals listed by citation counts.

Rank	Journal	Country	Counts
1	PEDIATRICS	USA	3033
2	ARCH OPHTHALMOL-CHI	USA	2991
3	OPHTHALMOLOGY	USA	2330
4	BRIT J OPHTHALMOL	ENGLAND	2181
5	INVEST OPHTH VIS SCI	USA	2174
6	NEW ENGL J MED	USA	2012
7	AM J OPHTHALMOL	USA	1777
8	J AAPOS	USA	1735
9	J PEDIATR-US	USA	1543
10	ARCH DIS CHILD-FETAL	USA	1498

### 3.2. Authors analysis and their cooperation

**Figure 4:**
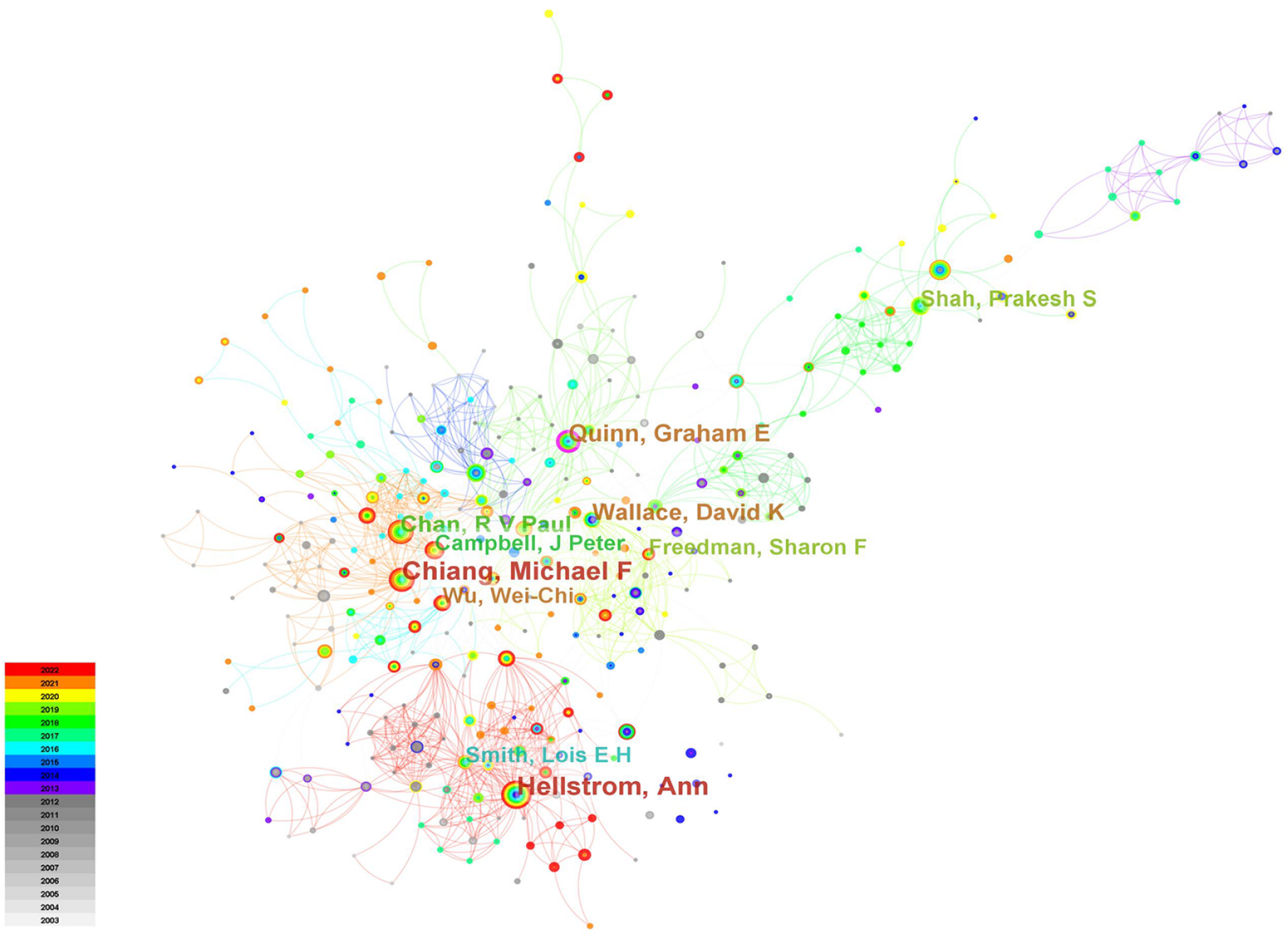
The author cooperation network on ROP.

Figure 4 showed the cooperation map of authors in ROP studies. Among them, the top 10 with highest number of publications were listed in Table 4. Hellström A was the most productive author with 99 papers published, followed by Chiang MF (*n* = 96) and Quinn GE (*n* = 65). The analysis of co-cited authors was also performed and presented in Table 4.

**Table 4:** The top 10 authors and co-cited authors.

Rank	Author	Publications	Rank	Co-Cited Author	Counts
1	Hellström, Ann	99	1	Good, William V	1317
2	Chiang, Michael F	96	2	Gole, Glen A	1101
3	Quinn, Graham E	65	3	Gilbert, Clare	813
4	Chan, R V Paul	58	4	Hellström, Ann	751
5	Wallace, David K	51	5	Smith, Lois E H	740
6	Freedman, Sharon F	49	6	Palmer, Earl A	685
7	Campbell, J Peter	46	7	Mintz-Hittner, Helen A	672
8	Shah, Prakesh S	46	8	Quinn, Graham E	548
8	Smith, Lois E H	42	9	Fierson, Walter M	501
10	Wu, Wei-Chi	42	10	Garner, A	461

### 3.3. Co-cited reference analysis

**Figure 5:**
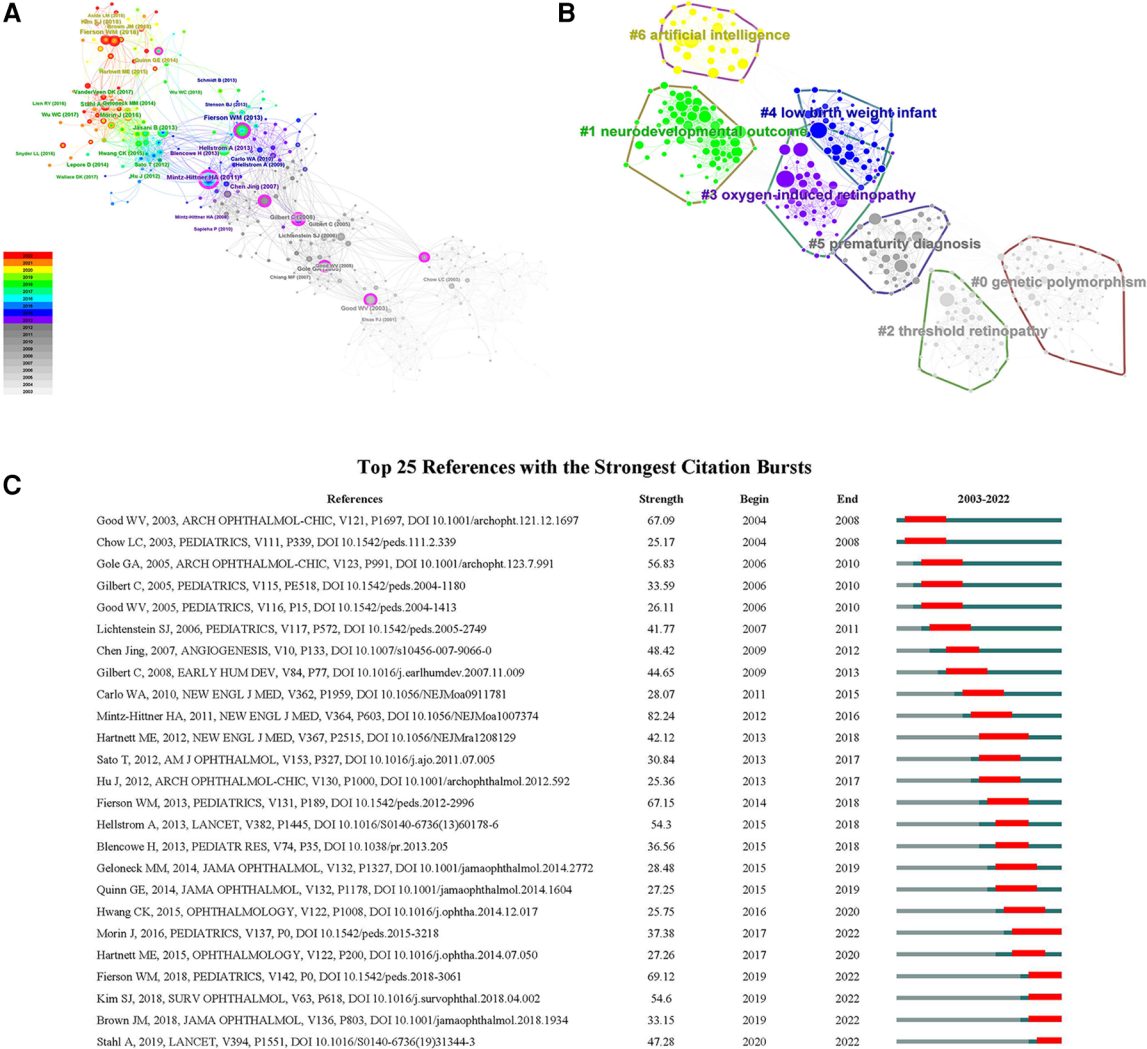
(**A**) The co-citation network of references; (**B**) the cluster map of co-cited references; (**C**) the top 25 references with the strongest citation bursts on ROP.

The co-citation network of references was shown in Figure 5A and consisted of seven major clusters (Figure 5B and Supplementary Table S1). The largest cluster (#0) was labeled as genetic polymorphism, followed by clusters neurodevelopmental outcome (#1), threshold retinopathy (#2) and oxygen-induced retinopathy (OIR) (#3). The low birth weight infant cluster (#4) and the prematurity diagnosis cluster (#5) possessed the same size of references. Finally, the 7th cluster (#6) was labeled as artificial intelligence (AI). After citation bursts detection, top 25 references were listed in Figure 5C. There remained five papers in burst phase until 2022. They were mainly related to the treatment outcomes of intravitreal injection and laser therapy (Morin J, 2016; Stahl A, 2019), screening examination (Fierson WM, 2018), risk factors (Kim SJ, 2018) and automated diagnosis with deep learning (Brown JM, 2018).

### 3.4. Keywords analysis and their evolutionary path

**Figure 6:**
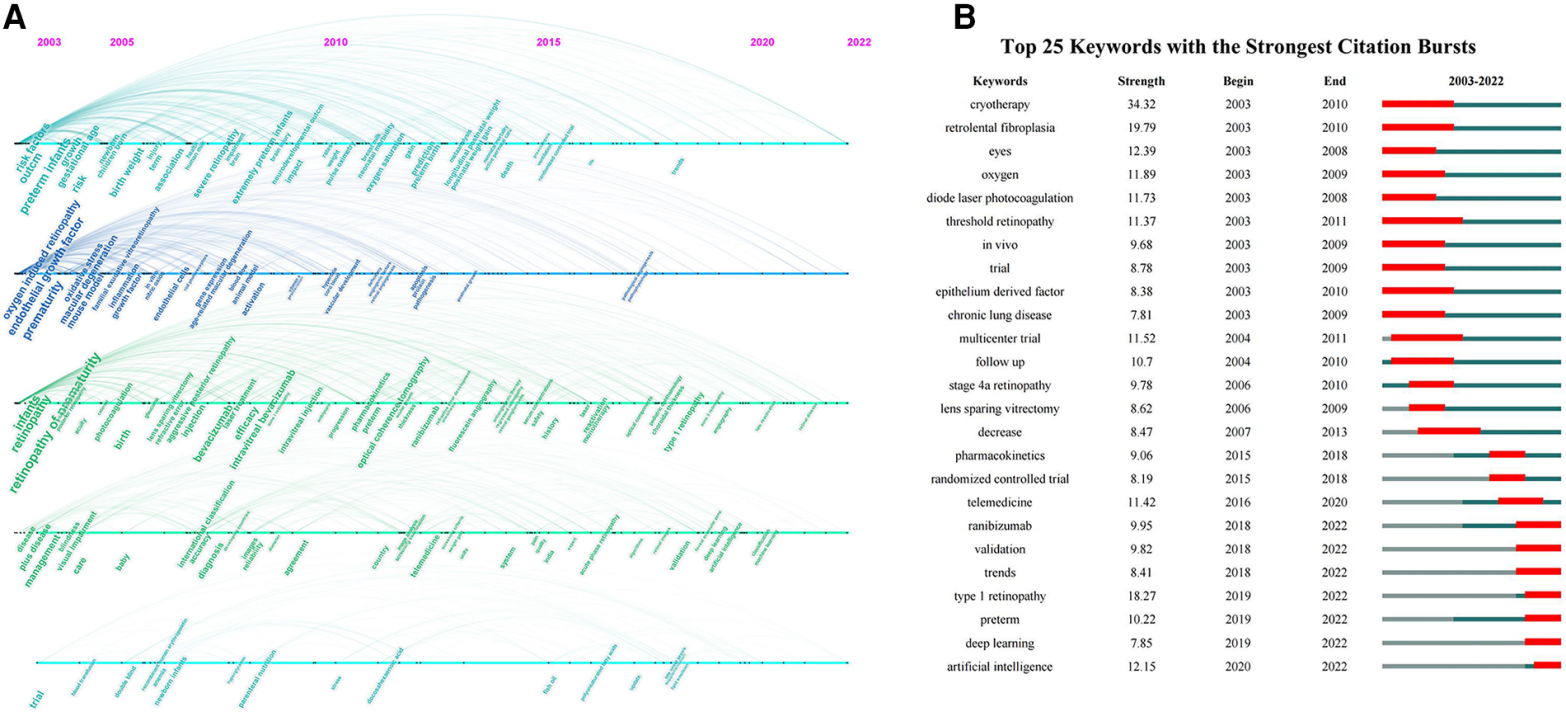
(**A**) The timeline visualization of keywords; (**B**) the top 25 keywords with the strongest citation bursts on ROP.

The keywords of ROP research were analyzed and displayed in a timeline (Figure 6A). Around 2005, studies mainly focused on the underlying mechanisms and risk factors of ROP, including gestational age, birth weight, oxygen and endothelial growth factor. In addition, the treatment outcomes of cryotherapy and laser photocoagulation were heatedly discussed. Between 2005 and 2010, new surgical methods gradually gained attention, especially intravitreal injection of anti-vascular endothelial growth factor (VEGF) and lens sparing vitrectomy. Between 2010 and 2015, with the advance of technology, optical coherence tomography and fundus fluorescein angiography (FFA) were gradually applied for ROP diagnosis and follow-up examination. Meanwhile, it provided the chance to learn more about the development of fovea and peripheral retina in ROP patients. Furthermore, the promotion of telemedicine contributed a lot to early screening with images and to alleviating healthcare burden. Around 2019 and 2020, the research direction turned to the interdisciplinary applications of AI which was trained to automatically diagnose or even predict ROP so as to help with clinical decision-making. Among the top 25 keywords with citation bursts shown in Figure 6B, AI and deep learning were still in their burst phase as of 2022, which meant that they were not only the current research hotspots but might also be the research trends in the coming years. The same went for “ranibizumab” and “type 1 retinopathy”.

## 4. Discussion

At present, ROP is still a difficult problem for ophthalmologists and pediatricians in neonatal diagnosis and treatment. Our study quantitatively and qualitatively analyzed the literature on ROP published from 2003 to 2022 at the levels of authors, countries, institutions, journals and keywords. Totally, it revealed seven major research hotspots after clustering the co-cited network of references, concerning pathogenesis, diagnosis, treatments and prognosis of ROP. In addition, among the top 25 keywords with the strongest citation burst, there remained seven bursting until 2022, suggesting they might still be the research hotspots in the following years.

### 4.1. Pathogenesis and risk factors

There are two major pathological phases in premature retina: cessation of normal vascularization and emergence of neovascularization (8). In phase I, preterm infants are in hyperoxia, partly because the room air provides higher oxygen saturation than uterus and partly because of the additional oxygen supplementation. At the same time, the concentration of growth factors, such as VEGF and insulin-like growth factor 1 (IGF-1), in infants decreases due to the loss of maternal supply, resulting in stop of vessel growth and hypoxia in retina. It was suggested that low levels of IGF-1 suppressed the VEGF-induced endothelial cell survival and was directly related to the ROP development (9). Subsequently in phase II, retinal hypoxia induces pathological vessel growth and even fibrous proliferation via a key mediator hypoxia-inducible factor-1α (HIF-1α) (10). Under normoxia, HIF-1α is unstable and continuously undergoes degradation in cells. However, in adaptation to hypoxic condition, HIF-1α is stabilized and then transferred into nuclei to activate the transcription of proangiogenic genes like VEGF, resulting in pathological neovascularization. On the other hand, oxidative stress also plays an important role in two pathological phases (11). Firstly in phase I, hyperoxia can induce the excessive production of reactive oxygen species, triggering the apoptosis of vascular endothelial cells and subsequent vascular obliteration. Then in phase II, hypoxia can induce the release of nitric oxide, upregulating the expression of proangiogenic factors. Therefore, oxygen is a major risk factor for ROP pathogenesis, and accordingly, most experimental studies related to ROP are conducted by using the “OIR” (cluster 3) model (12). Clinically, Srivatsa B et al. reported that infants of extremely low birth weight (BW) were more likely to develop severe ROP with higher fraction of inspired oxygen (13). However, lower oxygenation target could result in increased mortality in preterm infant, as the SUPPORT study group noted (14). Therefore, it is necessary to further explore an optimal range of oxygen saturation for preterm infants so as to minimize the incidence and severity of ROP while ensuring their life safety. At the same time, more investigations on antioxidants such as vitamin E and fish oil can be carried out for their potential benefits in ROP prevention (15, 16).

Also, the low gestational age (GA) and low BW (cluster 4) are important risk factors for ROP (17, 18). In order to timely identify and treat high-risk infants, different countries or regions have set their own screening standards based on GA and BW. For example, The US guideline recommended infants with BW ≤ 1,500 g or GA ≤ 30 weeks to receive ROP screening (19), comparing with BW less than 2,000 g or GA less than 32 weeks in China (20). Moreover, it was suggested that the slow postnatal weight gain was strongly associated with the increased risk of ROP, possibly due to the low level of IGF-1 in preterm infants (21). On this basis, several new algorithms were developed considering BW, GA or early weight gain to predict the development of ROP (22, 23).

Furthermore, another risk factor of concern is genetic polymorphism (cluster 0) because evidence supports that several genes may involve in the susceptibility to ROP (24). Cooke RW et al. confirmed a significant difference in VEGF −634 G→C genotypes in infants with treated ROP, suggesting excess VEGF production caused by genetic differences predisposed to ROP development (25). In addition, Hellström A et al. reported that the low level of IGF-1 due to defects in IGF-1 gene or IGF-1 receptor gene could be associated with increased risk of ROP (26). Therefore, further exploration in genetic polymorphism may provide new ideas for ROP prevention and treatment.

### 4.2. Classification and diagnosis

As for the “prematurity diagnosis” (cluster 5) of ROP, it mainly includes two aspects: the international classification and the digital imaging of retina. The International Classification of Retinopathy of Prematurity (ICROP) was first published in 1984 and most recently revised in 2021. At first, location of three zones, five stages of severity, plus disease and regressed ROP were defined for clinical description (27, 28). Then the 2005 version added the concept of pre-plus disease and aggressive-posterior ROP, which was later replaced by aggressive ROP (A-ROP) in 2021 (29, 30). At the same time, the definition of “reactivation” and “long-term sequelae” of ROP were supplemented. Moreover, in the CRYO-ROP study, there was another concept “threshold ROP” (cluster 2), which was referred to stage 3 ROP in zone 1 and zone 2 reaching at least 5 contiguous or 8 cumulative clock hours with plus disease (31). Based on this, prethreshold ROP was defined as any stage ROP in zone 1 and zone 2, stage 2 in zone 2 with plus disease and stage 3 in zone 2 but less than threshold. Later developed algorithms further classified prethreshold ROP into type 1 (including any stage in zone 1 with plus disease, stage 3 in zone 1 with or without plus disease, as well as stage 2 and stage 3 in zone 2 with plus disease) and type 2 (32).

Although the conventional binocular indirect ophthalmoscopy is the primary recommendation for screening examinations of ROP because of its higher sensitivity for mild lesions, it has the disadvantage of being costly and time-consuming for some areas (19, 33). Instead, digital retinal imaging is more cost-effective especially for telemedicine. It should be noted that the quality of images must be ensured before being interpreted by experienced ophthalmologists (34).

### 4.3. Treatments and prognosis

Nowadays, laser photocoagulation, anti-VEGF intravitreal injection and lens-sparing vitrectomy are three effective treatments for different stages of ROP. The ETROP study supported that peripheral laser treatment should be considered for threshold and type 1 ROP so as to prevent them from more unfavorable outcomes (35). From the long-term effect, Ospina LH et al. reported that the laser therapy enabled most patients to reach a satisfactory vision at age 5 or above (36). However, there is still a risk of disease progression or complications such as cataract and retinal scaring. As for type 2 ROP, conservative follow-up was recommended (35).

More recently, intravitreal injection of anti-VEGF drugs is an emerging treatment for acute ROP (37). First of all, studies supported that the treatment effectiveness was similar in bevacizumab, ranibizumab and conbercept (38, 39). Mintz-Hittner HA et al. discovered that compared to laser treatment, intravitreal bevacizumab contributed not only to the control of stage 3+ ROP in zone 1, but also to continuing growth of peripheral retinal vessels (40). In addition, the RAINBOW study reported that ranibizumab was effective in active ROP with fewer short-term complications and possibly better visual prognosis (41, 42). However, recurrence or even late retinal detachment could occur with anti-VEGF monotherapy because of the presence of peripheral avascular retina, which might require prophylactic laser therapy (43, 44). Thus, a close follow-up after anti-VEGF therapy is needed. On the other hand, there remains a discussion on the neurodevelopmental outcome (cluster 1) of anti-VEGF therapy. As VEGF is a neuroprotective factor capable to promote neural differentiation and repair damaged neurons, inhibition of VEGF activity can have a large effect on the development of the nervous system (45). Wu WC et al. found that intravitreal VEGF was able to enter the blood circulation, and therefore may cross the blood-brain barrier to impair the neurons of infants (46). Morin J et al. suggested that at 18 months’ corrected age, proportion of neurodevelopmental abnormalities assessed by neurologic examination and Bayley Scales in ROP infants treated with anti-VEGF was higher than that with laser (47). Similarly, Arima M et al. reported a neurodevelopmental delay evaluated by the Kyoto Scale of Psychological Development in ROP infants who received anti-VEGF treatments (48). However, neurodevelopmental delay itself is a common complication in preterm infants, which can be associated with cerebral damage, neonatal infection, oxygen load and visual impairment caused by ROP (49–52). As shown in a prospective study conducted by Fan YY et al, the Bayley III scores were similar in ROP infants at 1–3 years old with or without anti-VEGF treatment (53). So far it is not conclusive whether intraocular anti-VEGF therapy for ROP infants has long-term adverse effects on neonatal neurodevelopment. Therefore, a longer follow-up and a more comprehensive assessment in neurodevelopment are needed in future studies.

Finally, lens-sparing vitrectomy is recommended for stage 4 ROP to relieve the retinal traction (54, 55). Long-term follow-ups showed most patients could maintain useful visual outcomes and clear lens after surgery (56, 57). As for more severe retinal detachment, it may require combined vitrectomy, lensectomy and even scleral buckling, along with a limited prognosis (58–60).

### 4.4. Automated diagnosis and prediction

In recent years, deep learning, as an important algorithm in the branch of AI (cluster 6), is revolutionizing the medical field. In ophthalmology, convolutional neural network (CNN), U-Net and many other models are commonly used in computer-assisted detection of the images (61). Therefore, research initially focused on the automated diagnosis of ROP by using digital fundus images in screening. After employing the deep CNN models, Brown JM et al. trained an algorithm to automatically diagnose plus disease, while Huang YP et al. developed a system to detect stage 1 and stage 2 ROP (62, 63). To go a step further, Peng Y et al. proposed a fused network that was able to perform five-level staging (64). In addition to the fundus photographs, a deep learning network was applied to identify treatment-requiring ROP using FFA images (65). From the perspective of clinical application, studies showed that the AI-assisted image screening of ROP is both cost-effective and relatively reliable in results (66, 67).

Besides, AI has shown great potential in prediction of ROP. Previously, in order to further improve the sensitivity and specificity of ROP screening while reducing the costs, several research teams have developed algorithms to predict treatment-requiring ROP, such as the G-ROP criteria and CO-ROP algorithm (23, 68). However, these prediction models did not include the observation of retina, which can now be complemented by deep learning. For example, Wu Q et al. constructed a deep learning model combining fundus images and clinical information to predict the onset and severity of ROP (69). What’s more, by using artificial neural network, Huang CY et al. designed a predictive model of visual acuity for patients treated with ROP (70). In conclusion, the research prospect of AI-assisted ROP diagnosis and treatment is promising.

## 5. Strength and limitations

Our study summarized the research hotspots over the past two decades in the field of ROP and possible trends in the coming years by bibliometric analysis. However, there remains several limitations. Firstly, we did not include the literature published in 2023 on the date of data collection. Secondly, we only collected articles and reviews published in English from the WoSCC database, which might not be comprehensive enough to cover all the publications in this field. Finally, we only used the Citespace system for analysis, and the results may vary between different softwares.

## 6. Conclusions

Previous research hotspots of ROP have focused on the pathogenesis, diagnosis, treatment and prognosis. In addition to the conventional laser therapy, intravitreal injection of anti-VEGF drugs has been gradually applied in clinical practice and showed promising results. However, further exploration of the long-term effects is needed. On the other hand, automated diagnosis and prediction of ROP has highly attracted attention in recent years, which may provide strong support for clinical decisions in the near future.

## Data Availability

The original contributions presented in the study are included in the article/**Supplementary Material**, further inquiries can be directed to the corresponding authors.
